# Which is the best intrinsic motivation signal for learning multiple skills?

**DOI:** 10.3389/fnbot.2013.00022

**Published:** 2013-11-12

**Authors:** Vieri G. Santucci, Gianluca Baldassarre, Marco Mirolli

**Affiliations:** ^1^Laboratory of Computational Embodied Neuroscience, Isituto di Scienze e Tecnologie della Cognizione, Consiglio Nazionale delle RicercheRoma, Italy; ^2^School of Computing and Mathematics, University of PlymouthPlymouth, UK

**Keywords:** intrinsic motivations, learning signals, multiple skills, hierarchical architecture, competence acquisition, reinforcement learning, simulated robot

## Abstract

Humans and other biological agents are able to autonomously learn and cache different skills in the absence of any biological pressure or any assigned task. In this respect, Intrinsic Motivations (i.e., motivations not connected to reward-related stimuli) play a cardinal role in animal learning, and can be considered as a fundamental tool for developing more autonomous and more adaptive artificial agents. In this work, we provide an exhaustive analysis of a scarcely investigated problem: which kind of IM reinforcement signal is the most suitable for driving the acquisition of multiple skills in the shortest time? To this purpose we implemented an artificial agent with a hierarchical architecture that allows to learn and cache different skills. We tested the system in a setup with continuous states and actions, in particular, with a kinematic robotic arm that has to learn different reaching tasks. We compare the results of different versions of the system driven by several different intrinsic motivation signals. The results show (a) that intrinsic reinforcements purely based on the knowledge of the system are not appropriate to guide the acquisition of multiple skills, and (b) that the stronger the link between the IM signal and the competence of the system, the better the performance.

## 1. Introduction

The ability to learn and cache multiple skills in order to use them when required is one of the main characteristics of biological agents: forming ample repertoires of actions is important to widen the possibility for an agent to better adapt to different environments and to improve its chance of survival and reproduction.

Moreover, humans and other mammals (e.g., rats and monkeys) explore the environment and learn new skills not only on the basis of reward-related stimuli but also on the basis of novel or unexpected neutral stimuli. The mechanisms related to this kind of learning processes have been studied since the 1950s, first in animal psychology (e.g., Harlow, 1950; White, 1959) then in human psychology (e.g., Berlyne, 1960; Ryan and Deci, 2000), under the heading of “Intrinsic Motivations” (IMs). Recently, researchers have also started to investigate the neural basis of those mechanisms, both through experiments (e.g., Wittmann et al., 2008; Duzel et al., 2010) and computational models (e.g., Kakade and Dayan, 2002; Mirolli et al., 2013), and IMs are nowadays an important field of research (Baldassarre and Mirolli, 2013a).

From a computational point of view, IMs can be considered a useful tool to improve the implementation of more autonomous and more adaptive artificial agents and robots. In particular, IM learning signals can drive the acquisition of different skills without any assigned reward or task. Most of the IM computational models are implemented within the framework of reinforcement learning (Sutton and Barto, 1998) and, following the seminal works of Schmidhuber (1991a,b), most of them implement IMs as intrinsic reinforcements based on the prediction error (PE), or on the improvement in the prediction error (PEI), of a predictor of future states of the world.

Despite the increasing number of computational researches based on IMs (e.g., Barto et al., 2004; Schembri et al., 2007b; Oudeyer et al., 2007a; Santucci et al., 2010; Baranes and Oudeyer, 2013), it is not yet clear which kind of IM reinforcement signal is the most suitable for driving a system to learn the largest number of skills in the shortest time. To our knowledge, there are only few studies dedicated to this important issue (Lopes and Oudeyer, 2012; Santucci et al., 2012a, 2013b). In our previous works (Santucci et al., 2012a, 2013b), we have shown the importance of coupling the activity of the mechanism generating the IM signal to the competence of the system in performing the different tasks. However, in Santucci et al. (2012a) we limited our analysis to the learning of a single skill in a simple grid-world environment, while in Santucci et al. (2013b), although implementing a hierarchical architecture able to learn multiple tasks within continuous states and actions spaces, we focused only on signals based on PE. Lopes and Oudeyer (2012) deal with a similar problem, i.e., learning *n* tasks in the best possible way within a limited amount of time. The solution they propose is to allocate each unit of learning time to the task that guarantees the maximum improvement. However, their work tackles the problem in an abstract and disembodied setup and, moreover, they assume that the system has the information on the learning curves of each task.

In this work, we provide an exhaustive analysis of this scarcely investigated problem: which kind of IM reinforcement signal is the most suitable for driving the learning of skills in the shortest time. With this work we also aim to validate our hypothesis on the importance of a close coupling between the IM learning signal and the actual competence of the system in learning the different tasks. To this purpose, we implemented an artificial agent with a hierarchical architecture that allows the acquisition of several skills and we tested its performance in a setup with continuous states and actions, comparing both PE-based and PEI-based IM signals generated by different mechanisms. Some of the tested systems are taken from the computational literature related to IMs, including both the works of other researchers and our own; others derive from existing mechanisms but have not been tested before. The origin of each mechanism is indicated in section 2.3 where the different algorithms are explained in detail.

## 2. Materials and methods

### 2.1. The experimental setup and the simulated robot

**Figure 1:**
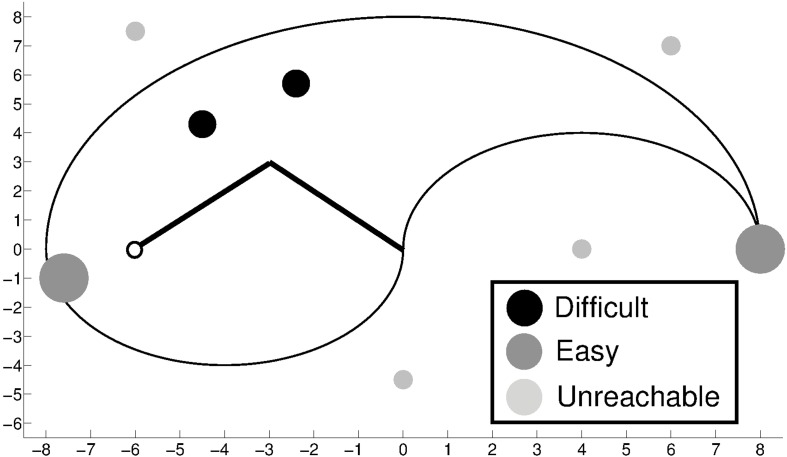
**The two dimensional work space of the simulated kinematic robotic arm with the target objects**. Small light-gray objects are unreachable by the arm.

The experimental task (Figure 1) consists in learning to reach for different circular objects positioned within the work space of a simulated kinematic robotic arm. The system has to learn in the best way and possibly shortest time a certain number of different skills, solely on the basis of IM reinforcement signals.

There are 8 different objects, corresponding to 8 different tasks: 2 are easy to be learnt, 2 are difficult and 4 are impossible to reach. The difficulty of the tasks is estimated on the basis of preliminary experiments where we tested the average time needed by a non-modular system to learn each of the different tasks with a performance of 95% (which is the average target performance in our experiments): easy tasks only need less than 2000 trials to be learnt while difficult tasks need more than 20,000 trials. Note that what we needed was not the precise measure of the difficulty of each task, but two classes of tasks differing substantially in the amount of trials needed to be learnt.

The choice of presenting tasks with different degrees of complexity derives from the evidence that an agent (be it an animal, a human, or a robot) can try to learn a great number of different abilities that typically vary considerably with respect to their learning difficulty, including many (probably the majority) that are not learnable at all (consider, for example, an infant trying to learn to reach for the ceiling). For this reasons, it is very important for a system to avoid trying to acquire unlearnable skills and to focus on those that can be learnt for the necessary amount of time (enough for a satisfying learning but no more than required).

The system is implemented as a simulated kinematic robot composed by a two degree-of-freedom arm with a “hand” that can reach for objects. The sensory system of the robot encodes the proprioception of the arm, i.e., the angles of the two joints. The output of the controller determines the displacement of the two joints in the next time step.

### 2.2. Arm controller and coding

**Figure 2:**
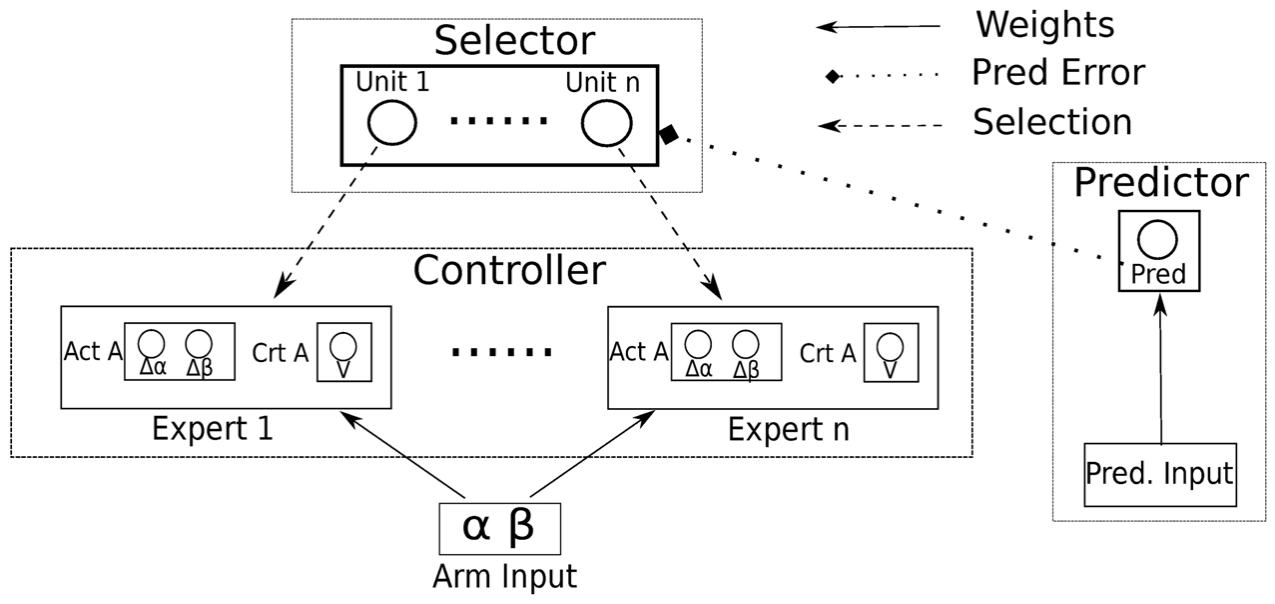
**The modular architecture of the system with the controller based on actor-critic experts, the selector and the predictor that generates the IM reinforcement signal driving the selector**. *n* is the number of the tasks; Act A is the output of the actor of the expert, controlling the displacement of the joints of the arm in the next step; Crt A is the evaluation made by the critic of the expert.

Since we are looking for a system able to learn different skills and cache them in its own repertoire of actions, we need an architecture where different abilities are stored in different components of the system (Baldassarre and Mirolli, 2013c). For this reason, the controller of the arm consists in a modular architecture (Figure 2) composed by *n* experts (8 in this implementation, one for each possible task) and a selector that determines which expert/task will be trained. For simplicity, we coupled each expert to a specific task so that the expert is reinforced only for reaching the associated object, but this assumption does not affect the generality of the results presented here.

Note that the values of the parameters in these experiments were chosen in different ways. The parameters of the experts are not directly connected to the goals of this work: here we are interested in which is the best IM signal for driving the acquisition of multiple skills regardless of the specific ability of the experts. For this reason, the parameters related to the experts are simply taken from our previous works (Santucci et al., 2010, 2013a; Mirolli et al., 2013). The parameters related to the selector and the selection procedure, as well as those connected to the reinforcement signal provided to the selector, derive from a hand search where we identified the values that guaranteed the best results. In particular, we isolated the crucial parameters (the learning rate of the predictors, the *temperature* of the *softmax* selection rule, and the temporal parameter α in the Q-learning rule that determines the activity of the unit of the selector: see below) and systematically (within limited ranges) changed their values in order to find a valid setup. Those that guarantee the best performance are the ones presented in the paper. Note that different values determine worse performances from a quantitative point of view (all the systems need more time to accomplish the tasks), but the differences between the experimental conditions are qualitatively stable.

Each expert is a neural network implementation of the actor-critic architecture (Barto et al., 1983) adapted to work with continuous state and action spaces (Doya, 2000). The input to the experts are the actual angles of the two joints of the arm, α and β (ranging in [0, 180]), coded through Gaussian radial basis functions (RBF) (Pouget and Snyder, 2000) in a two dimensional grid (10 × 10 units).

The evaluation of the critic (*V*) of each expert is a linear combination of the weighted sum of its input units. The actor of each expert has two output units, fully connected with the input, having a logistic transfer function:

(1) $$o_{j}=\Phi \text{​}\left(b_{j}+\sum_{i}^{N}w_{ji}a_{i}\right)\;\;\;\;\;\Phi (x)=\frac{1}{1+e^{-x}}$$

where *b*_*j*_ is the bias of output unit *j*, *N* is the number of input units, *a*_*i*_ is the activation of unit *i* and *w*_*ji*_ is the weight of the connection linking the input unit *i* to the output unit *j*. Each motor command *o*^*m*^_*j*_ is determined by adding noise to the activation of the relative output unit:

(2) $$o_{j}^{m}=o_{j}+q$$

where *q* is a random value uniformly drawn in [−0.1; 0.1]. The resulting commands are limited in [0, 1] and then remapped in [−25, 25] and control the displacement of the related arm joint angles.

In each trial, the expert that controls the arm is trained through a TD reinforcement learning algorithm. The TD-error δ is computed as:

(3) $$\delta =\left(R_{e}^{t}+\gamma_{k}V^{t}\right)-V^{t\;-\;1}$$

where *R*^*t*^_*e*_ is the reinforcement for the expert *e* at time step *t*, *V*^*t*^ is the evaluation of the critic of the expert at time step *t*, and γ is a discount factor set to 0.9. The reinforcement is 1 when the hand touches the object associated with the selected expert, 0 otherwise.

The connection weight *w*_*i*_ of critic input unit *i* is updated in the standard way (Sutton and Barto, 1998):

(4) $$\Delta w_{i}=\eta^{c}\delta a_{i}$$

where η^*c*^ is a learning rate, set to 0.08.

The weights of each actor are updated as follows (see Schembri et al., 2007a):

(5) $$\Delta w_{ji}=\eta^{a}\delta \left(o_{j}^{m}-o_{j}\right)\left(o_{j}\left(1-o_{j}\right)\right)a_{i}$$

where η^*a*^ is the learning rate, set to 0.8, *o*^*m*^_*j*_ − *o*_*j*_ is the discrepancy between the action executed by the system (determined by adding noise) and that produced by the controller, and *o*_*j*_(1 − *o*_*j*_) is the derivative of the logistic function.

The selector of the experts is composed by *n* units, one for each expert/task to be selected/learnt. At the beginning of every trial the selector determines the expert controlling the arm during that trial through a *softmax* selection rule (Sutton and Barto, 1998). The probability of unit k to be selected (*P*_*k*_) is thus:

(6) $$P_{k}=\frac{exp\frac{Q_{k}}{\tau }}{\sum_{i=0}^{n}exp\frac{Q_{i}}{\tau }}$$

where *Q*_*k*_ is the *Q*-value of unit *k* and τ is the *temperature* value that rescales the input values (here the *Q*-values) and so regulate the noise of the selection.

The activity of each unit is determined by a *Q-learning* rule used to cope with n-armed bandit problems with non-stationary rewards Sutton and Barto (1998):

(7) $$Q_{k}^{t+1}=Q_{k}^{t}+\alpha [R_{s}^{t}-Q_{k}^{t}]$$

where *Q*^*t*^_*k*_ is the *Q*-value of the unit corresponding to the selected expert during trial *t*, α is a temporal parameter set to 0.35 and *R*^*t*^_*s*_ is the reinforcement signal obtained by the selector.

The reinforcement signal (*R*^*t*^_*s*_) driving the selection of the experts is the intrinsic reinforcement that we want to analyse in order to find the one that is the most suitable for autonomously learning multiple skills. Such signal is based on the error, or the improvement in the error, of a predictor of future states of the world. We now consider the different signals compared in this work.

### 2.3. IM signals and predictors

#### 2.3.1. Prediction error signals

**Figure 3:**
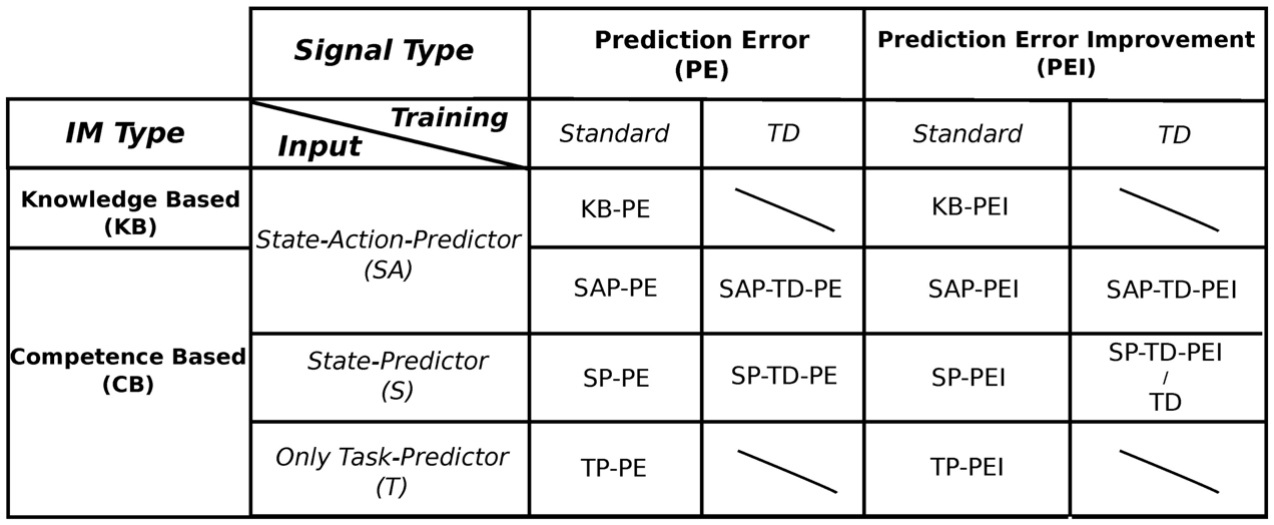
**Scheme of the different experimental conditions, divided by typology of signal, typology of intrinsic motivations, input, and training algorithm**. Note that the random (RND) condition is not mentioned in this table because it does not use any reinforcement signal to determine the selection of the experts. See Section 2.3.1 and 2.3.2 for a detailed description of all the different conditions.

As mentioned in section 1, we tested the IM signals and the mechanisms (predictors) implemented to generate such signals that are most used in the literature on IMs (see Figure 3 for a scheme of the different experimental conditions).

- **Knowledge-Based Predictor (KB-PE):** The first IM reinforcement signal was the prediction error (PE) of a predictor of future states of the world (Schmidhuber, 1991a): in this model, the IM signal is represented by the absolute value of the error in predicting future states. The proposed mechanism was based on a forward model receiving the actual state and the planned action as input and predicting the next state. The idea is that the system, driven by the intrinsic PE signal, would explore the environment looking for new states that are not predictable by the forward model, acquiring at the same time the competence in new skills related to those states.

However, such predictors generate a signal which is coupled to the knowledge of the mechanism (learning the model of the world) and not to the competence of the system (learning skills). This signal can be considered as a purely knowledge-based prediction error (KB-PE) IM signal which may turn out to be inadequate for driving the acquisition of a repertoire of skills (see Santucci et al., 2012a; Mirolli and Baldassarre, 2013). In order to provide a stronger link between the predictor and the competence of the system, an effective solution is to change the target of the predictions. Instead of trying to anticipate every possible future configuration, the predictor has to anticipate only one particular state, the one connected to the trained skill, i.e., the *goal state*. In this way the PE signal is generated on the basis of the error in predicting the achievement of the goal, i.e., the generation of the final result of the skill that the agent is learning. Unlike KB-IM, this kind of signals can be considered competence-based (CB) IM signals and the predictors that generate them can be identified as CB-IM mechanisms (for the distinction between KB-IM and CB-IM, see also Oudeyer and Kaplan, 2007b).

Here we tested different CB-IM mechanisms. While all these mechanisms learn to predict the achievement of the goal state, they differ in the information received as input. Note that all the predictors also receive the information on which expert/task is currently trained by the system.

- **State-Action Predictor (SAP-PE):** This predictor has the same input as KB-PE mechanism, that is the actual state (the two joints of the arm, α and β) and the planned action (Δα and Δβ), coded through RBFs. Training follows a standard delta rule. Examples of SAP-PE can be found in Santucci et al. (2010, 2013b).
- **State Predictor (SP-PE):** The SP-PE is not widespread in the literature. A similar predictor can be found in Barto et al. (2004), although this work proposed a system implemented within the option theory framework (Sutton et al., 1999), where the focus is more on the learning of the deployment of previously acquired skills rather than on the learning of the skills themselves. In our previous works (Santucci et al., 2012a, 2013b) we found that because its input is composed only by the actual state of the agent this kind of predictors are more closely coupled to the competence of the system than the SAP-PE: SP-PE mechanism is able to anticipate the achievement of the goal only when the agent has learnt the correct actions from the different states. Input is coded through RBFs. SP-PE is trained through a standard delta rule.
- **Temporal Difference SAP (SAP-TD-PE):** This predictor has the same input as SAP-PE but it is trained through a TD-learning algorithm with a discount factor set to 0.99. The implementation of this mechanism derives from the knowledge acquired in previous works (Mirolli et al., 2013; Santucci et al., 2013a) where we found that standard SAP-PE predictors do not work well with continuous states and actions. Providing the predictors with a TD algorithm solves some of these problems (for a generalization of TD-learning to general predictions, see Sutton and Tanner, 2005).
- **SP-TD-PE:** As for the SAP-TD-PE mechanisms, this predictor is the TD-learning version of SP-PE.
- **Task Predictor (TP-PE):** This predictor is inspired by our work in a simple grid-world scenario (Santucci et al., 2012a). A similar mechanism is implemented also in Hart and Grupen (2013). Differently from all the previous predictors, TP-PE does not make step-by-step predictions but a single prediction, at the beginning of the trial, on the achievement of the selected task. The input of this predictor consists only of the task/expert that has been selected, encoded in a *n*-long binary vector, with *n* equal to the number of tasks. The predictor is trained through a standard delta rule. These characteristics should provide a complete coupling between the signal generated by the predictor and the competence of the system in achieving each task: the predictor has no further information and can learn to anticipate the achievement of the target state only when the agent has really acquired a high competence in the related skill. In this way the selector should give the control to an expert only when it is effectively learning, shifting to a different expert when the competence to perform the related task has been completely acquired.

All CB-PE mechanisms generate a prediction (P) in the range [0, 1] related to the expectation that the system will accomplish the goal state within the time out of the trial. The error in predicting the goal state provides the intrinsic reinforcement signal to the selector of the system, whose activity determines which expert controls the system during the next trial and, at the same time, determines the expert that is trained by the system. This PE reinforcement signal is always positive: with the KB mechanism it is equal to the absolute value of the error; with CB mechanisms it is 1-P when the system reach the goal state and 0 otherwise.

For all the systems implemented with the different PE mechanisms, the *temperature* τ value of Equation 6 is set to 0.01.

#### 2.3.2. Prediction error improvement signals

As pointed out by Schmidhuber (1991b), PE signals may encounter problems in stochastic environments: if the achievement of a target state is probabilistic, the predictor will continue to make errors indefinitely. This means that the reinforcement will be never completely canceled and the system may keep on trying to train a skill even when it cannot improve any more. In order to solve this problem several systems (e.g., Schmidhuber, 1991b; Oudeyer et al., 2007a) use the improvement of the prediction error (PEI) rather than the PE as the IM signal.

For this reason, we also tested all the mechanisms described in section 2.3.1 using their PEI (instead of the PE) as the reinforcement signal for the selector. Examples of KB-PEI can be found in Schmidhuber (1991b); Huang and Weng (2002); Baranes and Oudeyer (2009); an example of a SAP-PEI mechanism can be found in Oudeyer et al. (2007a). All the other mechanisms (SP-PEI, SAP-TD-PEI, SP-TD-PEI and TP-PEI), are tested here for the first time.

The PEI at time *t* was calculated as the difference between the average absolute PEs calculated over a period *T* of 40 time steps:

(8) $$PEI_{t}=\frac{\sum_{i=t-(2T-1)}^{t-T}{\left|PE\right|}_{i}}{T}-\frac{\sum_{i=t-(T-1)}^{t}{\left|PE\right|}_{i}}{T}$$

In addition to the other mechanisms, in the PEI condition we also tested another CB-IM signal (Schembri et al., 2007a,b; Baldassarre and Mirolli, 2013b):

- **Temporal-Difference Predictor (TD)**: This mechanism uses the TD-error (see Equation 3) of the selected expert as the intrinsic reinforcement signal that drives the selector. More precisely, here we use the average TD-error within the trial as the IM signal. Indeed, the TD-error can be considered a measure of the expert improvement in achieving its reinforcement and for this reason a measure of the competence improvement.

For all the systems implemented with the different PEI mechanisms the *temperature* τ value of the Equation 6 is set to 0.008. For the TD mechanism, the *temperature* τ is 0.01, while the α of Equation 7 is 0.25.

In order to better evaluate the performance of the simulated robot in the experimental setup when driven by the IM signals generated by the different mechanisms, we also tested a system that selects experts randomly (RND). Sometimes random strategies can indeed turn out to be surprisingly good: however, the best IM signal to drive the selection and acquisition of different skills in the shortest time, should guide the system better than a random selection.

### 2.4. Hypotheses and comparative criteria

The main purpose of this work is to investigate which is the most suitable IM learning signal for driving the acquisition of a repertoire of different skills in the shortest time. In our previous works (Santucci et al., 2012a, 2013b), we proposed that the most important feature of such a signal should be its coupling with the competence in the skill that the system is trying to learn. For this reason our first hypothesis is that competence based signals should perform better then knowledge based ones.

With respect to the various CB mechanisms implemented, we expect that the TD versions of SAP and SP conditions should perform better than their normal versions since we know from previous works (Mirolli et al., 2013; Santucci et al., 2013a) that the latter ones do not work well with continuous states and actions. Furthermore, we also expect TP to perform better than both SAP and SP. With respect to PE vs. PEI, we predict that PE signals may behave a bit better than PEI signals, as the latter are probably more noisy and less strong than the former. Finally, we do not know how the TD error signal may perform with respect to the other PEI signals.

We compare the different IM signals by measuring their velocity in learning multiple tasks. In particular, we run different experiments (see section 3) and count the number of trials (averaged over several repetitions of the experiment) needed by each condition to achieve an average performance of 95% in the 4 learnable tasks. We chose the average of 95% as the target performance since we want a value that is able to identify a satisfying capability of a system to learn different skills. If we used a different target performance (e.g., 90 or 99%) they would be qualitatively the same.

## 3. Results

Each condition was tested for 400,000 trials. At the beginning of every trial the selector determines which expert will control the activity of the arm in that trial. Each trial ends if the selected expert reaches its target object or after a time out of 20 time steps.

For every mechanism, we ran different simulations varying the learning rate (LR) of the predictor (9 different values) because we wanted to be sure that the results were not dependent on the use of a specific set of LRs. For each LR we ran 20 repetitions of the experiment. In the TD and RND condition, where there is not a separate predictor (in RND there is no IM signal, in TD we use the TD-error of the experts), we ran 180 repetitions of the experiment to balance the total number of replications in the other conditions.

### 3.1. PE signals

**Figure 4:**
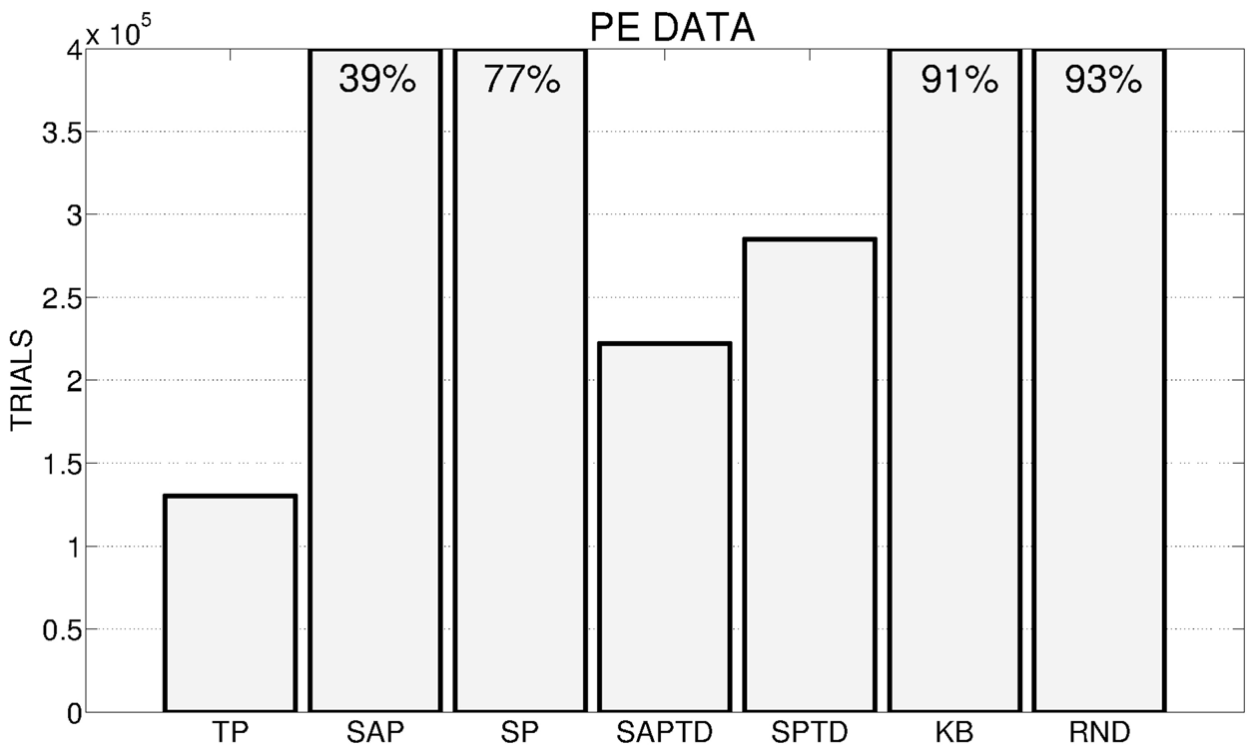
**Average number of trials needed by the different conditions to achieve an average performance of 95% in the 4 learnable tasks (average results of 180 replications: 20 replication by 9 learning rates for the systems with predictors, 180 replications for the random system) in the different experimental conditions**. If a system has not reached 95% at the end of the 400,000 trials we report on the corresponding bar the average performance at the end of the simulation.

Figure 4 shows the number of trials (averaged over the 180 replications) needed by the different PE conditions to achieve an average performance of 95% in the 4 learnable tasks. The results clearly underline, confirming one of our hypotheses, how the TP-PE mechanisms is the one that generates the best signal to drive the system in achieving a high average performance in the learnable tasks in the shortest time (average of about 130,000 trials). As expected (see Section 2.3.1 and 2.4), the SAP-PE and the SP-PE are not able, working within continuous states and actions, to generate a good signal to guide the selection and the learning of skills. SAP-TD-PE and SP-TD-PE are able to drive the system in achieving the average target performance within the 400,000 trials but they are slower than the TP-PE system. Both KB-PE and RND conditions can reach high performance within the end of the experiment (more than 90%), but they are not able to achieve the target value of 95%. An interesting result is that the system driven by the random selection reaches an average performance (93%) higher than the one driven by KB-PE mechanism (91%).

**Figure 5:**
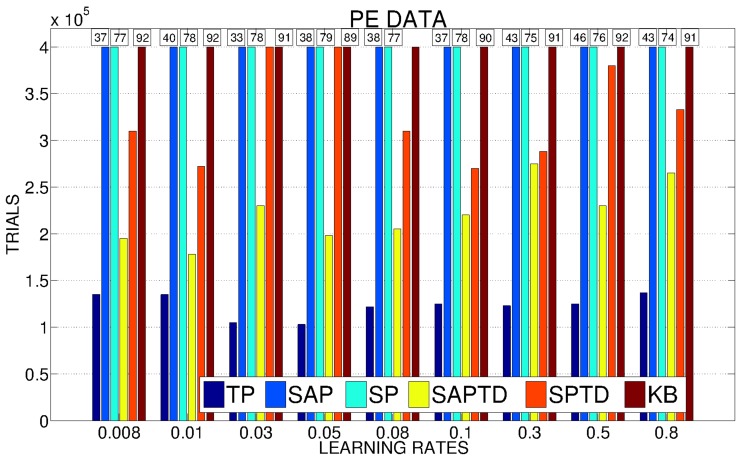
**Average number of trials needed by the system to achieve a performance in the 4 learnable tasks of 95% with different values of the learning rates of the predictors (average on 20 replications per learning rate)**. If a system has not reached 95% we report above the corresponding bar the average performance at the end of the simulation.

In Figure 5 we show a detailed analysis of the average performance of the system in the different conditions with different values of the learning rate for the predictors. SAP-PE and SP-PE are not able, regardless from the learning rate of the predictor, to achieve the target performance, while SAP-TD-PE and SP-TD-PE seems to be sensitive to the value of the learning rate of the predictor (SP-TD-PE more than SAP-TD-PE). Differently, TP-PE is very robust with respect to the value of the learning rate of the predictor: regardless of this value this condition is always the best performer, being able to achieve a high performance in a short time.

**Figure 6:**
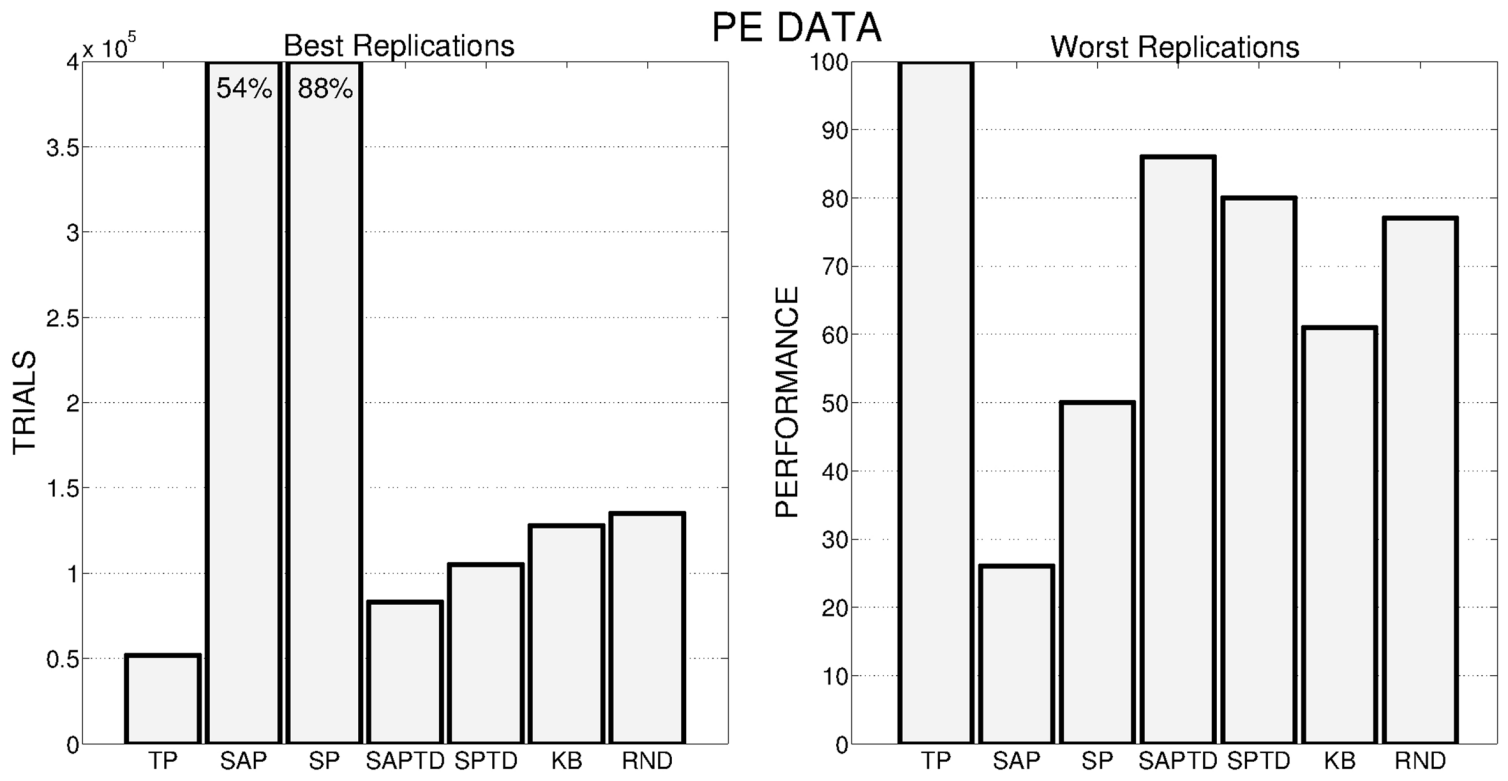
**Left**: Number of trials needed by the best replication of each condition to achieve the target performance. When the target value is not achieved within the time limit, the final performance is reported inside the bar. **Right**: Average performance achieved by the system in the worst replication of each experimental condition.

These general results are even more evident if we look at Figure 6, where the performance of the best and worst replications of every condition are shown: the overall best performance is achieved by a replication of the TP-PE condition that is able to reach the target performance in about 50,000 trials. As in the case of average performances, the best replications of SAP-PE and SP-PE are not able to reach the target performance while KB-PE and RND have comparable performance. Even more impressive are the results of the worst replications: the TP-PE mechanism is the only one that is able to drive the system in achieving the target performance within the given time also in its worst replication. The other conditions reflect the average results, with the KB-PE condition performing worse than random selection in its worst replication.

To understand the causes of these results, for each condition we analyzed the average selections of the experts connected to the 4 learnable tasks during time and the average level of performance achieved on those tasks. Data are related to the best learning rate value of the predictor of each different condition. In this way we can check if the signal generated by the predictors is able to drive the selector in a proper way, following the actual competence acquired by the experts. Data of RND system are not shown: in this case experts are always selected (on average) uniformly, and hence the system wastes time in selecting experts that cannot learn anything or that have already learnt their tasks (e.g., the two easy tasks).

**Figure 7:**
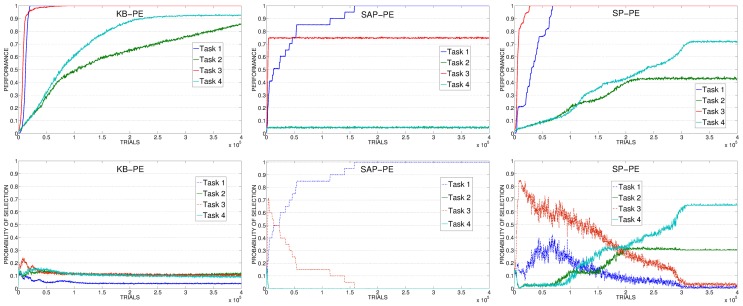
**Top:** Average performance on the 4 learnable tasks in the best condition (with respect to the learning rate of the predictor) of KB-PE, SAP-PE, SP-PE. **Bottom:** Average selection probability for the experts associated to the 4 learnable tasks, in the same condition.

Figure 7 (left) shows the results of the KB-PE mechanisms. In this condition the system is not driven by an IM signal connected to the competence of the system in learning the skills, but to the knowledge acquired by the predictor in anticipating every possible future state. For this reason the system is not selecting the experts connected to the tasks that are still to be learnt, but rather the experts that are surprising the predictor reaching whatever unpredicted state. These experts include also those related to the 4 non-learnable tasks. This process leads to a random selection (random-selection value is 0.125 because it is calculated on all the 8 tasks). While this is not a problem for the two easiest tasks (task 1 and task 3) that are learnt after few trials, the canceling of the IM signal and the consequent absence of a focused learning severely impairs the learning of the difficult tasks (task 2 and task 4).

The result of the KB-PE condition confirm one of our main hypotheses, clearly underlining how a KB-IM signal is inadequate to properly drive an agent in learning different skills: it either continues to select already learnt tasks, or it does not properly select those that are still to be learnt. This is the reason why, if we are looking at improving the competence of a system, we should use CB-IM mechanisms.

If we look at data related to SAP-PE and SP-PE (Figure 7, center and right) it is clear that these mechanisms are not able to cancel in a proper way the PE signal provided by the achievement of the goal states. For this reason SAP-PE, on average, focuses on one of the easiest tasks (whose target states, on average, are rapidly discovered by the system) although the robot has completely acquired the related competence. SP-PE is able to anticipate the achievement of the easy tasks, but it learns too slowly these predictions: for this reason, although task 1 and task 2 have both been learned at about 70,000 trials the system still focuses on them for further trials, wasting precious time for learning the more difficult skills.

**Figure 8:**
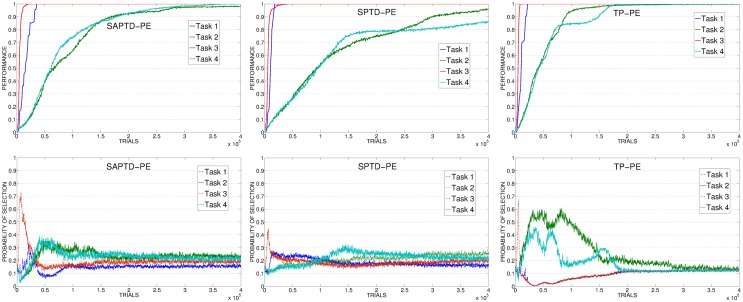
**Top:** Average performance on the 4 learnable tasks in the best condition (with respect to the learning rate of the predictor) of SAP-TD-PE, SP-TD-PE, TP-PE. **Bottom:** Average selection probability for the experts associated to the 4 learnable tasks, in the same conditions.

SAP-TD-PE and SP-TD-PE (Figure 8, left and center) present the opposite problem: these mechanisms learn very fast to predict the reaching of the objects, even faster than the actual competence of the system in those tasks. Although these are CB mechanisms, the learning process of these predictors is not strictly coupled with the ability of the system to reach for the objects. This is evident comparing the progress in the performance with the selections: the predictors cancel the signals before the system has acquired the competence related to the different tasks determining a selection which is not optimally coupled to the actual performance of the system. However, in spite of this problem, these mechanisms are able to guide the system in reaching the target performance within a reasonable time. This is because, differently from KB-PE and RND, although turning too fast to a random selection, they perform selections only on the 4 learnable tasks (that are the only ones that can generate a PE) and not on all the 8 tasks. SAP-TD-PE and SP-TD-PE do not provide a perfect IM signal, but they are a good example of how even a sub-optimal CB-IM signal is able to drive the learning of skills better than a KB signal.

Differently from all the other conditions, the TP-PE mechanism (Figure 8, right) is able to drive the complete learning of the skills in relatively few trials. The reason of this performance is connected to the signal generated by the TP-PE mechanism: this signal is strictly coupled with the competence of the system in the task that it is learning. Looking at the average development of the experiment, it is clear how the selector, driven by this CB-IM signal, assigns the control of the robot only to an expert connected to a task that has still to be learnt, shifting to another one when a skill has been fully achieved. Easy skills need just few trials to be learnt and for this reason the system focuses on their training (and selection) only for a very short time at the beginning of the experiment. As soon as the predictor has learnt to anticipate the achievement of those target states, it cancels their respective signals and drives the agent to search for other skills to acquire. Difficult tasks require a longer time to be learnt so the system focuses on selecting the related experts longer, until a high performance has been achieved. When all the tasks have been learnt the predictor has learnt to anticipate the achievement of all the target states, so the selector receives no more intrinsic reinforcements and generates an (almost) random selection.

### 3.2. PEI signals

**Figure 9:**
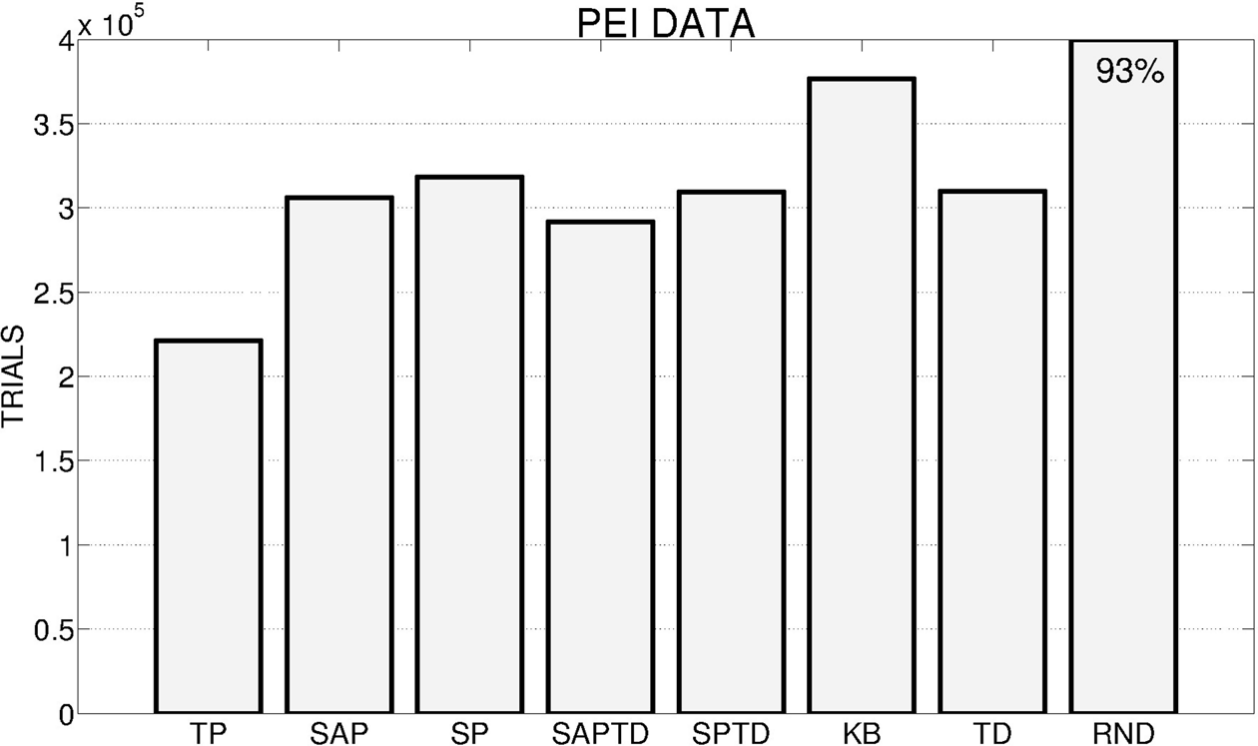
**Average number of trials needed by the system to achieve an average performance of 95% in the 4 learnable tasks (average results of 180 replications: 20 replications by 9 learning rates for the systems with predictors, and 180 replications for the RND ad TD conditions) in the different experimental conditions**. If a system has not reached 95% we report on the corresponding bar the average performance at the end of the simulation.

Figure 9 shows the average number of trials needed by the system to achieve the target performance of 95% within the different conditions. As with the PE signal, also with the PEI signal the TP-PEI condition is the one that is able to guide the system in achieving the target performance in the shortest time. However, the average number of trials needed by those conditions that best perform with PE signals (TP, SAP-TD, SP-TD) is raised. At the same time, those conditions that with PE signal were not able to achieve the target average performance (95%) in the learnable tasks, with PEI significantly improve their results, with SAP-PEI and SP-PEI reaching a performance similar to SAP-TD-PEI and SP-TD-PEI. This is due to the properties of PEI signal: if a predictor is not able to improve its ability to anticipate the achievement of a target state, there is no improvement in the prediction error and the signal is canceled. So, despite the predictor is not able to correctly anticipate the achievement of the easy tasks even when their competence is fully acquired (as in SAP-PEI and SP-PEI conditions), the constant error generates no PEI signal and allows the system to shift to the selection of different experts possibly discovering new learnable skills. The TD condition guarantees a performance that is similar to those of the other CB signal (except for TP-PEI, which is the best performer), while when the system is driven by the KB-IM signal it is not able to achieve satisfying results: KB-PEI turns out to be the worst PEI condition.

**Figure 10:**
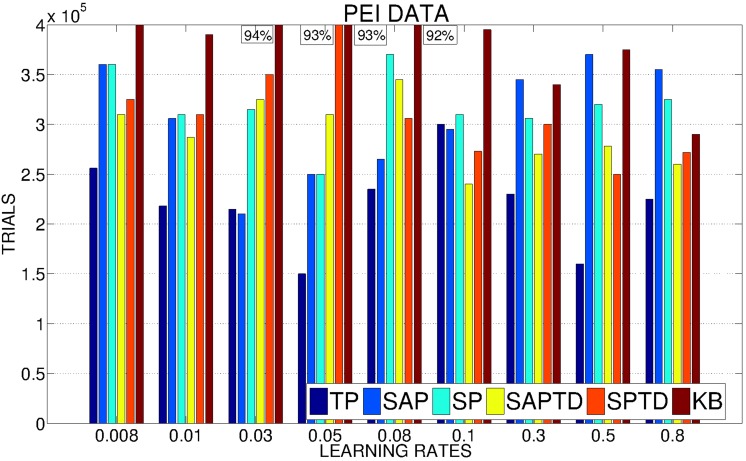
**Average number of trials needed by the system to achieve a performance in the 4 learnable tasks of 95% with different values of the learning rates of the predictors (average on 20 replications per learning rate)**. If a system has not reached 95% we report above the corresponding bar the average performance at the end of the simulation.

As anticipated in our hypotheses, PEI signals are much noisier and weaker than PE signals. This is clear from Figure 10, showing how all the conditions (including TP) present a high sensitivity to the variation in the learning rate of the predictors. However, TP-PEI is the one that is able to drive the system in achieving the target performance in the shortest number of trials (only 150,000, on average, with learning rate 0.05).

**Figure 11:**
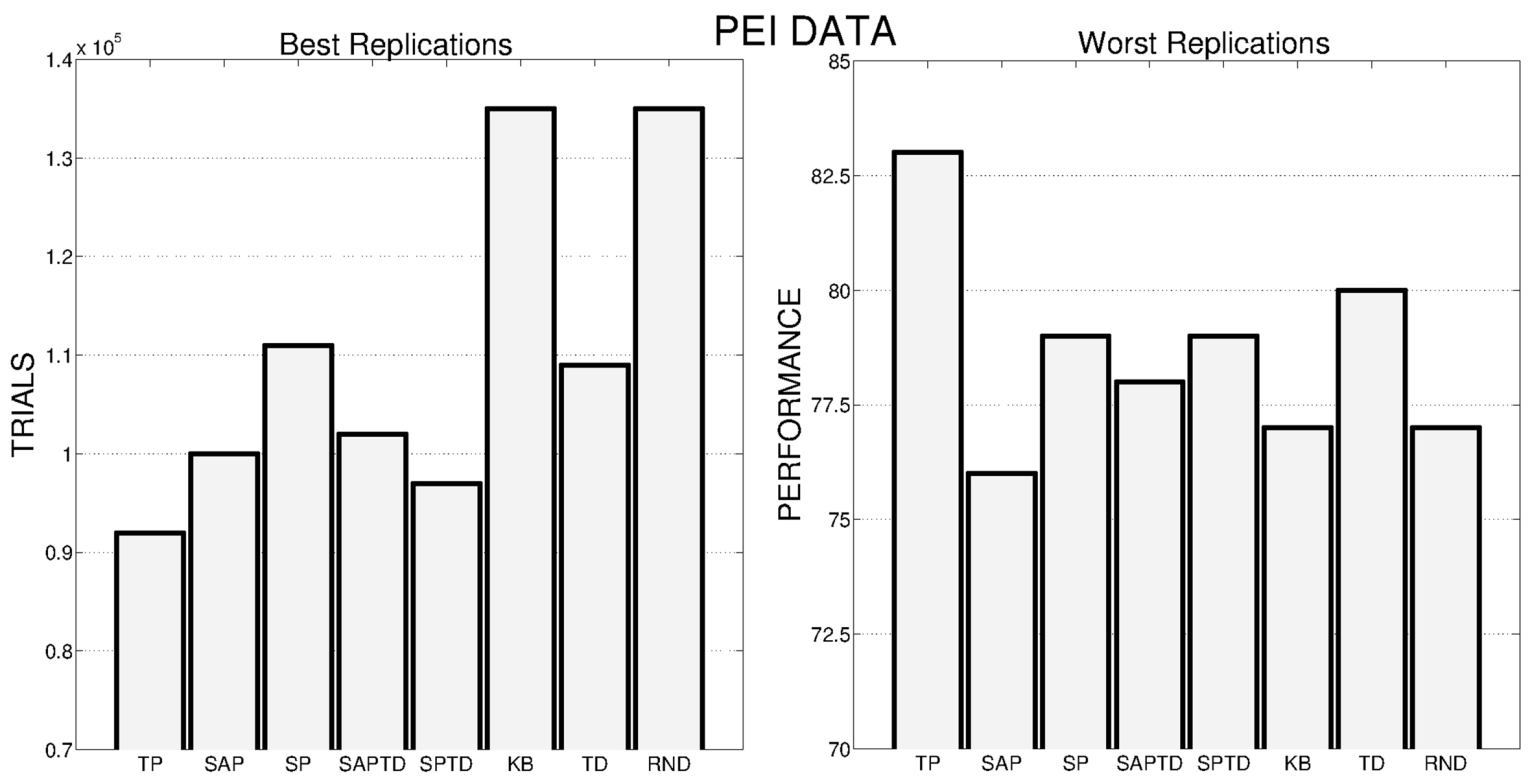
**Left:** Number of trials needed by the best replication of each condition to achieve the target performance. **Right:** Average performance achieved by the system in the worst replication of each experimental condition.

Data on the average performances are confirmed by Figure 11, where we show the best (Figure 11, left) and worst (Figure 11, right) replications of all the different conditions. As for PE signal, also with PEI the best replication of the TP-PEI condition is the absolute best among all the replications of all the conditions and even its worst replication is the one that reaches the highest performance compared to the worst replications of the other conditions. KB-PEI confirms to be the worst PEI condition: even its best replication (Figure 11, left) is performing as the RND selector. TD condition shows a great variance in its different replications: its best replication (Figure 11, left) is only the 5th performer, while its worst replication is the second best (among the worst replications of all the conditions) after the TP.

As with PE experiments (section 3.1), to better understand the results we analyzed data showing the average selections of the experts connected to the 4 learnable tasks during time and the average level of performance achieved on those tasks. Data are related to the best learning rate value of each different condition, while for TD condition we look at the average performance and selections on 20 replications (consecutive and including the best replication of the condition).

**Figure 12:**
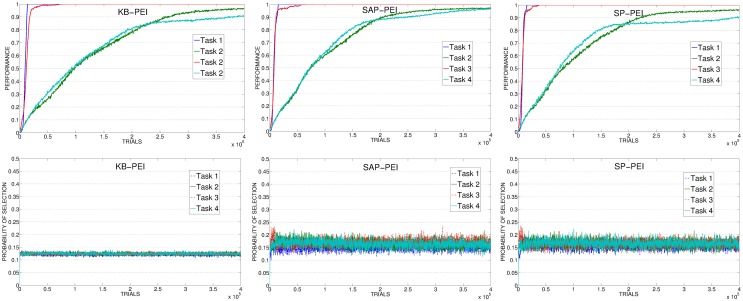
**Average performance on the 4 learnable tasks (top) and average selection probability for the associated expert (bottom) in the best condition (with respect to the learning rate) of KB, SAP, SP**.

The poor performance of KB-PEI (Figure 12, left) is related to the bad selection determined by the KB-IM signal: the experts related to the 4 learnable tasks are clearly selected randomly.

When driven by CB-IM signals the system reaches a better performance, with differences between the conditions implemented with different mechanisms. In SAP-PEI and SP-PEI conditions the selection is very noisy (Figure 12, center and right). Although learnable tasks are selected more than in RND and KB conditions, the already weak signal is flattened by the activity of the predictors that are not able to significantly improve in their ability to anticipate the target states.

**Figure 13:**
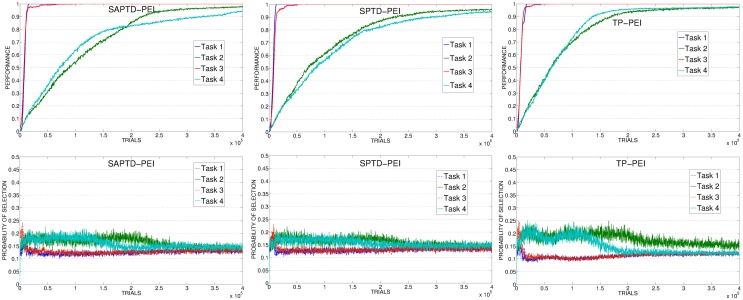
**Average performance on the 4 learnable tasks (top) and average selection probability for the associated expert (bottom) in the best condition (with respect to the learning rate) of SAP-TD-PEI, SP-TD-PEI, TP-PEI**.

SAP-TD-PEI and SP-TD-PEI (Figure 13, left and center) are able to cancel the signal deriving from the rapidly learnt easier tasks, but at the same time they present the problem we found with the PE: these mechanisms can be too fast in canceling the IM signal, determining a decrease in the probability of selecting the complex tasks even if the system has still competence to acquire. This is confirmed by looking at data of SAP-TD-PEI condition, where the PEI signal for task 4 is drastically decreased around 200,000 trials, when the system has reach an average performance on that task of only about 80%.

As in the experiment with the PE signal, the TP-PEI mechanisms is the one that is able to drive the system in selecting and learning the different skills in the shortest time. The reason is the same as with PE results: even in its PEI version, the CB-IM signal generated by the TP mechanism is the only one that is closely connected to the competence acquired by the system in the different learnable tasks (Figure 13, right). Easy tasks, which are learnt very fast, are selected only during the short time needed to raise their performance. Thanks to the canceling of the intrinsic reinforcement signal provided to the selector, the system is able to shift to the complex tasks. At about 150,000 trials, on average, the system has reached a high performance on task 4: due to the connection of the TP mechanism to the competence of the agent, the PEI-IM signal related to that task fades away and the system focuses only on the skill that at that time of the experiment is the least efficient (task 2).

**Figure 14:**
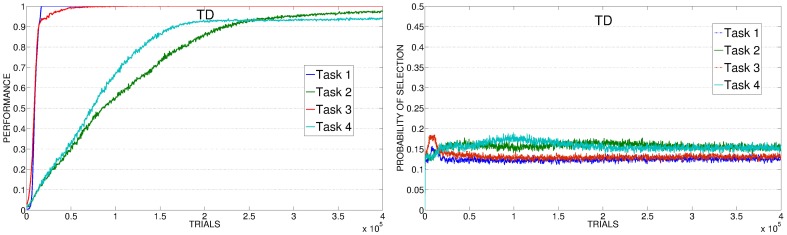
**Average performance and selections in TD condition**.

As mentioned in section 2.3.2, together with the different PEI signals we also tested another CB-IM signal provided by TD-error of the selected expert. As previously described, the average performance of TD condition is similar to those of other CB-IM conditions with PEI signal (except for TP, which is the best performer). However, if we look at the average performance on 20 replications (consecutive and including the best replication of this condition) we can see that when driven by the TD signal the system reaches a performance that is similar or even better than those of the other conditions (except for TP) in their best learning rate condition (confront Figure 14, left, with Figures 12, 13, top). Indeed, if we look at the average selections (Figure 14, right), we can see that TD signal is able to generate a sequence of selections that are connected to the competence progress of the system, although less than the one provided by the TP mechanism.

## 4. Discussion

In this paper we analyzed different kinds of IM signals in order to find the most suitable to drive a system in selecting and learning different skills in the shortest time. To tackle this important issue, we implemented a simulated two-dimensional kinematic robotic arm with a hierarchical architecture able to train and cache different skills and we tested it within continuous spaces and actions in an experimental scenario where the agent had to learn to reach different objects.

The first important result validate one of our main hypotheses: a purely KB-IM signal (as those implemented in Schmidhuber, 1991a,b; Huang and Weng, 2002) is not able to satisfactorily drive the acquisition of multiple skills. This signal is coupled to the knowledge of the KB predictor that tries to anticipate every possible future state of the world. The PE or PEI signal deriving from this kind of mechanism drives the system in exploring the environment without any specific target: this is why the performance of the KB condition is similar to RND condition, where the system is guided by a random selection of its experts. Note that the implementation provided in this work helps the KB mechanisms. Indeed, here we used the intrinsic reinforcement signal to drive the selection of the experts. In a previous work (Santucci et al., 2012a), we showed that if the KB-IM signal is provided directly to an actor-critic expert the system continues to explore the environment to train the predictor without learning any skill. With our results we are not saying that KB-IM are useless or wrong: simply they are involved in different processes, which are related to knowledge acquisition more than competence acquisition.

**Figure 15:**
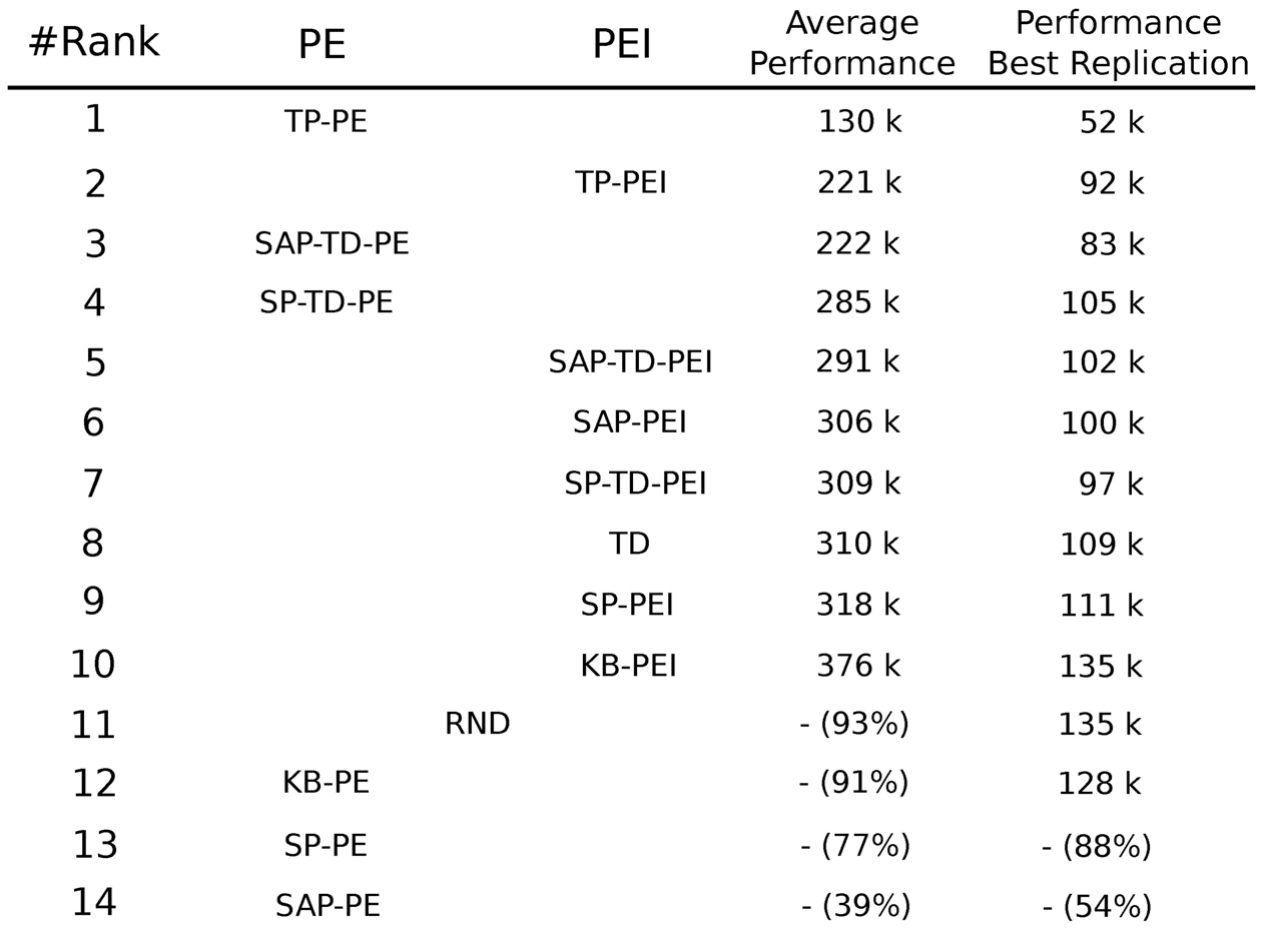
**Ranking of the different experimental conditions summarizing the result of both PE and PEI signals with respect to the ability to reach the target average performance of 95% in the four learnable tasks**. For every condition the performance of the best replication is also shown. Performances are measured in thousands of trials. If a condition has not reached 95% at the end of the 400,000 trials of the experiment we report the average performance at the end of the simulation.

In order to optimize the IM-based acquisition of skills, learning signals have to be strictly connected to the actual competence in those skills, i.e., to the actual competence in achieving target goals. CB-IM signals provide such a coupling and the results of our experiments underlie how the stronger that coupling, the better the performance of the system (see Figure 15 for the ranking of the results of all the experimental conditions). Indeed, not all the CB-IM mechanisms guarantee the same close connection between the correctness of the predictor and the competence acquired by the system. Some mechanisms like SAP and SP (especially when generating a PE signal) are not good predictors in continuous spaces and actions as they are *too slow*: they are not able to properly cancel the IM signal even if the agent has fully acquired the related competence, thus leading the system to focus on already trained experts. Other CB mechanisms (SAP-TD, SP-TD) turned out to provide a useful learning signal for the acquisition of skills, although they present the problem of being too fast in canceling the intrinsic reinforcement signal that fades away before the robot has completely learnt the related skills.

As expected, the condition that was able to learn all the skills in the shortest time, both in PE and PEI conditions, was the one where the IM reinforcement signal for the selector was generated by what we called TP mechanism: a predictor of the goal states (the target states connected to the different skills) that receives as input only the information on which expert has been selected to be trained. The mechanism that we proposed provides a close connection between the ability of the predictor in anticipating future target state and the actual competence acquired by the agent in the related skill. This coupling guarantees an IM signal which is particularly appropriate for the selection and acquisition of different skills: the intrinsic reinforcement is present when the system is learning a new task, it is canceled when the competence on that task has been learnt and reappears when a new, still-to-be-learnt task is encountered by the system.

Moreover, we also tested the TD condition where the TD-error signal of the active expert is used as the intrinsic reinforcement for the selector. This solution (Schembri et al., 2007a,b; Baldassarre and Mirolli, 2013b) is able to cope with the same problems connected to stochastic environments that may lead to use PEI signals instead of PE signals. The TD condition performs comparably to the other sub-optimal CB-IM driven conditions in PEI experiments. However, in its best replications, it is able to reach very high performance and, moreover, it presents important computational advantages: the absence of a separate component for the predictions reduces computational time and avoids the setting of its specific learning rate.

Despite the growing theoretical understanding of the differences between functions and mechanisms of IM (e.g., Oudeyer and Kaplan, 2007b; Stout and Barto, 2010; Santucci et al., 2012a; Mirolli and Baldassarre, 2013), their implications have not been fully exploited in specific models. In particular, there is still a confusion between KB mechanisms and CB mechanisms. Some still use KB-IM signals to drive the acquisition of competence, leading to inappropriate learning signals as underlined by the results of our present work. Others shifted, without realizing, to CB mechanisms probably because they encountered the problems connected to KB signals and competence acquisition. However, due to the lack of understanding of the differences between KB-IM and CB-IM, they turn out to implement sub-optimal CB mechanisms. An example is Oudeyer et al. (2007a) where, although they describe the implemented intrinsic signal as the PEI of the knowledge of the system, they use the predictor to anticipate few (three) high-level abstract important states (visual detection of an object; activation of a biting sensor; perception of an oscillating object). These high-level states represent few relevant states among a huge number of non-interesting states, and each of them can be achieved only with sequences of actions. This predictor is very similar to the SAP we tested in our experiment, which in fact is a CB mechanism, even if its results are not the best possible.

Looking at the implementation of our system, a strong limit is the fact that the possible tasks to be learnt are given at the beginning of the experiment. A further step toward more autonomous and versatile agents would be to built systems that self-determine their goals. Recently, some effort has been made in the field of hierarchical reinforcement learning to find good solutions to the problem of setting useful goals. Most of these techniques (e.g., McGovern and Barto, 2001; Mehta et al., 2008; Konidaris and Barto, 2009) focus on searching adequate sub-goals on the basis of externally given tasks (reward functions). Only few works (e.g., Mugan and Kuipers, 2009; Vigorito and Barto, 2010) tried to implement systems able to set their own goals independently from any specific task, which is a fundamental condition for real open-ended autonomous development.

Another important point concerns the generality of our results. In future works it will be interesting to test the different IM learning signals in different experimental setups (e.g., adding more dimensions and degrees of freedom; using a dynamic arm) where different and possibly more difficult tasks have to be learnt: this would be a further confirmation of our results and conclusions. However, we believe that the main findings of this work are quite general. Indeed, the differences between KB-IM and CB-IM lie in the typology of information used to determine such signals and not on the specific setups they are implemented in. Similarly, the conclusion that a proper CB-IM mechanism has to generate a signal which is closely connected to the actual competence of the system is a general finding that can be exploited regardless of the particular architecture used to implement the agent.

Our expectation is that testing the different IM signals studied here in more realistic conditions will strengthen the advantages of using the TP signal with respect to the other implementations of IMs. In a real environment the number of skills that can be acquired is much larger then the one considered here, and the difficulty to learn the skills is much more heterogeneous. Moreover, in the real world there are strong dependencies between different competences, so that some skills can be learnt only after learning others. All these characteristics of real environments emphasize the importance for an IM signal to be strongly connected to the competence of the system, thus avoiding to waste time in easy (or previously learnt) tasks or in too difficult (or not possible) tasks, and focussing on the skills that can be learnt at the moment, which may be later exploited to learn other skills. Our results show that only a signal that is closely linked to the competence of the system is able to provide these general features.

Looking at the different typologies of IMs, our intuition is that they may play complementary roles, with KB-IM being able to inform the system of novel or unexpected states of the environment, driving the agent to generate new target states, and CB-IM being able to guide the acquisition of the skills related to those targets. This further model, that tries to integrate the different typologies of IMs, will probably require a more complex architecture able to manage both the control of the effectors, the generation and selection of the different motivations and the combination of different IM learning signals.

### Conflict of interest statement

The authors declare that the research was conducted in the absence of any commercial or financial relationships that could be construed as a potential conflict of interest.
